# Assertive community treatment for elderly people with severe mental illness

**DOI:** 10.1186/1471-244X-10-84

**Published:** 2010-10-19

**Authors:** Jolanda Stobbe, Niels CL Mulder, Bert-Jan Roosenschoon, Marja Depla, Hans Kroon

**Affiliations:** 1 Research Centre O3, Erasmus mc, University medical center, Department of Psychiatry, PO Box 2040 Dp-0122, 3000 CA Rotterdam, the Netherlands; 2 BavoEuropoort, Centre for Mental Health Care, Rotterdam, the Netherlands; 3 Municipal Public Health Service, Rotterdam Rijnmond Division of Public Mental Health Care, the Netherlands; 4 VU University Medical Centre Amsterdam, Institute for Research in Extramural Medicine, Department of Nursing-Home Nedicine, the Netherlands; 5 Trimbos institute, Netherlands Institute of Mental Health and Addiction, Utrecht, the Netherlands

## Abstract

**Background:**

Adults aged 65 and older with severe mental illnesses are a growing segment of the Dutch population. Some of them have a range of serious problems and are also difficult to engage. While assertive community treatment is a common model for treating difficult to engage severe mental illnesses patients, no special form of it is available for the elderly. A special assertive community treatment team for the elderly is developed in Rotterdam, the Netherlands and tested for its effectiveness.

**Methods:**

We will use a randomized controlled trial design to compare the effects of assertive community treatment for the elderly with those of care as usual. Primary outcome measures will be the number of dropouts, the number of patients engaged in care and patient's psychiatric symptoms, somatic symptoms, and social functioning. Secondary outcome measures are the number of unmet needs, the subjective quality of life and patients' satisfaction. Other secondary outcomes include the number of crisis contacts, rates of voluntary and involuntary admission, and length of stay. Inclusion criteria are aged 65 plus, the presence of a mental disorder, a lack of motivation for treatment and at least four suspected problems with functioning (addiction, somatic problems, daily living activities, housing etc.). If patients meet the inclusion criteria, they will be randomly allocated to either assertive community treatment for the elderly or care as usual. Trained assessors will use mainly observational instruments at the following time points: at baseline, after 9 and 18 months.

**Discussion:**

This study will help establish whether assertive community treatment for the elderly produces better results than care as usual in elderly people with severe mental illnesses who are difficult to engage. When assertive community treatment for the elderly proves valuable in these respects, it can be tested and implemented more widely, and mechanisms for its effects investigated.

**Trial Registration:**

The Netherlands National Trial Register NTR1620

## Background

Adults aged 65 plus are a fast-growing segment of the Dutch population. This sharp rise is leading to an increase in the number of elderly people who live on their own and who have psychiatric disorders [1,2]. Just 8%-16% of elderly people with such disorders receive treatment from a healthcare provider [1,3,4]. This group of older psychiatric patients is not only difficult to engage, but often also has a range of serious problems in other aspects of their lives [2]. Some of them neglect themselves and their immediate surroundings, and thus live in extreme squalor [5,6]. Exact numbers of (self)-neglected elderly are not known [5,6]. But elderly in the community with depressive symptoms or cognitive impairment are at risk for development of self-neglect [7] and more than half of the elderly who are neglecting their environment has a psychiatric disorder such as dementia, paranoid disorder, addiction or depression [6]. It is unknown whether mental healthcare services to date are sufficient to help them, e.g. because these patients drop out of care due to the lack of motivation, [8,9], or whether active outreach care, such as assertive community treatment (ACT), is needed.

In adult mental healthcare, ACT is a much-examined, frequently used organizational model for treating difficult-to-engage patients with severe mental illness (SMI; 10, 11). Particularly in the United States, ACT has been proven to be effective in reducing admissions and making patients' housing status more stable. It improves patients' satisfaction and motivation for treatment [12,13]. A relatively high number of ACT patients also remain in care [14,15]. We think this last observation is very important, since drop out of care may lead to further deterioration.

European studies, however, have been less unanimous about the effects of ACT and showed no difference between ACT and care as usual (CAU) in terms of psychosocial functioning and hospital admission days [16-18]. Part of this discrepancy may be explained by improvements in the quality of CAU in Western Europe [19,20], but also by the fact that CAU contains some features of ACT [21].

Intervention studies for SMI elderly in the community are rare. Two studies showed that intensive community care given to depressive elderly people (sometimes with delusions) caused a fall in the number of admissions to a psychiatric unit, stabilization of psychiatric symptoms, and an improvement in quality of life [22,23] as compared to CAU. Another study showed that psychiatric symptoms and functioning improved when elderly people with psychiatric problems received outreach services rather than office-based ones [3]. We found no studies that focused on the effects of ACT for patients aged 65 and over.

### Research aim

Using a randomized controlled trial (RCT) design, we will compare the effects of ACT elderly (ACTE) with those of CAU for elderly patients with SMI who are difficult to engage in care.

### Hypotheses

Our first hypothesis is that ACTE will decrease the number of patients who drop out of care (number of patients who have no registered contact with the services over a period of 3 months and/or the number of patients discharged out of care). The other primary hypotheses include that ACTE will increase the number of patients engaged in care (patients who receive care within 3 months after admission to the team) and will improve patients' psychiatric, somatic and social symptoms. Secondary hypotheses include that ACTE reduce the number of unmet needs more than CAU does. We also expect an increase in patients' subjective quality of life and in patients' satisfaction with mental-health care. Finally we hypothesize that to a greater extent than CAU, ACTE will reduce the number of crisis contacts, reduce voluntary and involuntary admission rates and reduce length of stay in a psychiatric hospital.

## Methods - Design

This trial is being funded by BavoEuropoort centre for mental healthcare, Rotterdam, the Netherlands.

### Research design

This RCT will use one intervention group consisting of patients who receive treatment according to ACTE, and one control group consisting of patients who receive treatment according to CAU. We will use a pre-randomized block design, with four patients per block, whereby patients are randomized *before *they consent to participate, the so-called Zelen's design [24]. The design and execution of this study were approved by the Dutch union of medical-ethic trial committees for mental health organizations

### Participants - setting

The study was carried out in the Netherlands by BavoEuropoort centre for mental healthcare in the greater Rotterdam area (1.3 million residents). BavoEuropoort has 1,300 staff, who are employed at 32 sites in eleven municipalities. It provides treatment and guidance to people in whom complex psychiatric disorders are combined with problems in several life domains. It has various outpatient clinics and clinical settings for voluntary or involuntary admission. There are six ACT teams for patients who are difficult to engage in treatment. One of these teams focuses on elderly patients. BavoEuropoort also provides mental healthcare services for elderly people (55+) in their third and fourth stages of life who have mental health complaints and/or cognitive disorders.

Participants are elderly outpatients (aged 65 years or older) with severe psychiatric problems, who are difficult to engage, and who are resident in the BavoEuropoort catchment areas.

### Procedure

BavoEuropoort has one ACT team for elderly people (intervention condition). In the control condition (CAU), participants were identified by three general community mental health teams working for elderly people living in the community. Two of these teams focused on patients with psychiatric disorders; the other focused mainly on patients with cognitive impairments. Community mental healthcare professionals from these teams will use a checklist to determine whether all new elderly service-users meet the inclusion criteria. The inclusion of patients started July 2008 and was closed on 1 August 2010.

**Figure 1:**
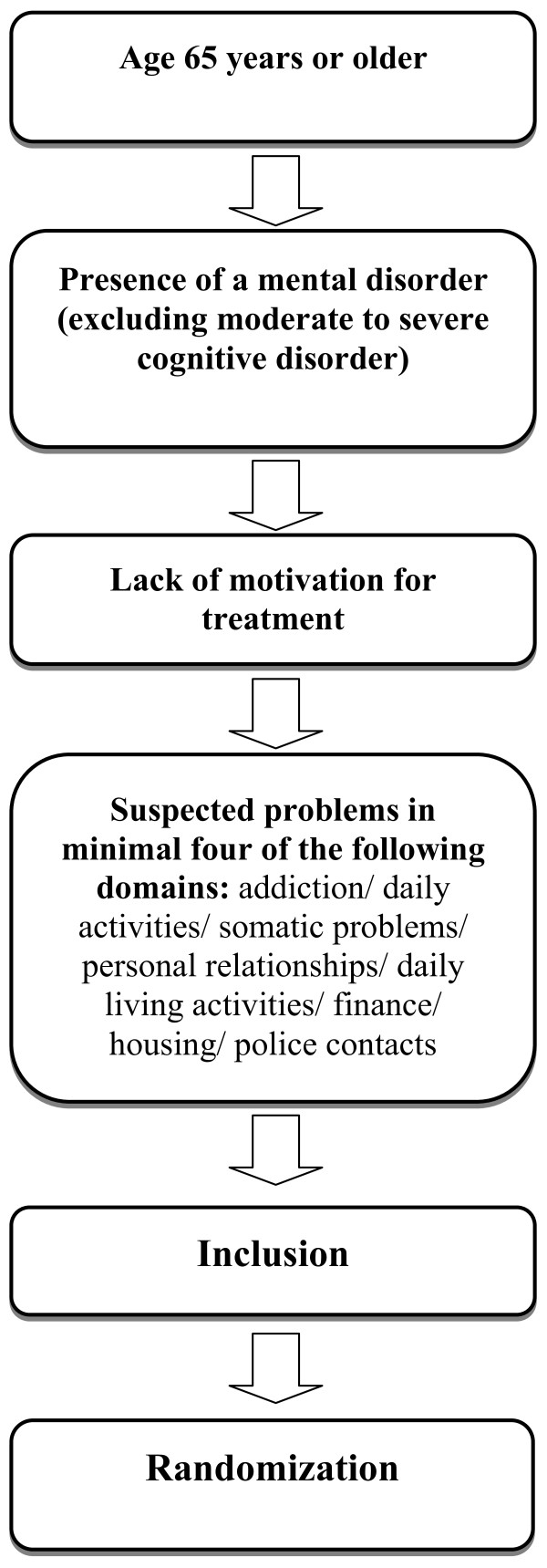
**Inclusion flowchart**.

Figure 1 shows the inclusion flowchart; inclusion criteria are:

1. age 65 year or older;

2. having a severe mental illness (patients with a severe psychiatric disorder, usually psychotic or bipolar disorder or severe depression, leading to a complex combination of psychiatric and social needs).

3. no motivation for treatment (actively or passively resisting treatment; the patient is very difficult to involve in any form of treatment, including treatment by the general practitioner).

4. and 4 problems of the following domains:

- addiction: taking alcohol and drugs taking not in a social context, occasional minimal loss of control of drinking or drugs use or adverse consequences or incapacitated due to alcohol or drug problems.

- somatic problems: all kind of somatic problems, including illness or disability from any cause that limits mobility, impairs sight or hearing, or otherwise interferes with personal functioning.

- daily living activities: the overall level of functioning in activities of daily living: e.g., problems with basic activities of self-care such as eating, washing, dressing, toilet; also complex skills such as budgeting, recreation and use of transport, etc.

- housing: the overall severity of problems regarding the quality of living conditions, accommodation and daily domestic routine, taking into account of the patient's preferences and degree of satisfaction with such circumstances. Are the basic necessities met?

- daytime activities: the overall level of problems regarding the quality of the daytime environment. Limited or no daytime activities. The patients problems are made worse by a lack of daytime activities.

- social relationships: problems associated with social relationships, as identified by the patient or apparent to carers or others. These included active or passive withdrawal from social relationships, a tendency to dominate, social relationships or non-supportive, destructive or self-damaging relationships.

- finances: problems associated with finance (such as debts), and problems with skills such as budgeting.

- police contacts: contact within police in the last year that were a product of the patients psychological situation. Examples: nuisance, public drunkenness or minor offence by psychological situation.

We exclude patients with severe cognitive problems (severely disorientated, for example consistently disorientated with regards to time to time, place, or person, or suffering memory impairment for example only fragments remain, loss of distant as well as recent information, unable to effectively learn any new information, no effective communication possible through language or inaccessible to speech.

Primary and secondary outcome variables were collected by means of an interview of patients willing to give informed consent as well as from the patients' files. A trained independent research assessor will record these primary and secondary outcome measures. This research assessor contacts after patient randomization the clinician to make an appointment to visit the patient together. In this contact with the patient the research assessor asked questions (via semi structured interview) to rate the assessment instruments. Because patients are difficult to engage they don't fill in questionnaires themselves. If patients refuse to answer questions, the research assessor will collect data from the patient's records or via asking the clinician about the patients' situation. Data are collected as part of the routine outcome assessments of the mental health centre. The first interview was done closely after inclusion in the study. The second interview took place 9 months after the first and the last interview after 18 months.

### Intervention and care as usual

The intervention will be ACTE, an integrated multidisciplinary treatment model. It will have four main components: 1.) the caseload will be both small and shared (10 patients per case manager), 2.) services and assertive outreach will all be community based, 3.) all services (i.e. both medical and psychosocial) will be provided by the ACTE team, and 4.) and all services will be provided on an unlimited basis [25-27].

ACTE has been described in a manual that describes the target group, the team's working method, and tasks of the various disciplines involved. It also includes various programmes (modules) based on other evidence-based practices. Under ACTE, care is given with regard to various essential life domains, at whichever location the patient is [28]. Professional mental healthcare workers invest in a long time relationship with the patient, even if he or she initially continues to refuse care.

To address the most common problems of the target group, the ACTE team will represent several disciplines (table 1), and will have ten full-time equivalents working force. The low caseload will allow for intensive contact with patients. Team members will collaborate closely on each patient, using one treatment plan.

**Table 1:** Differences between ACTE and CAU

Intervention Group (ACTE)	Control Group (CAU) Gerontology psychiatry teams	Control Group (CAU) Psycho geriatric team
A shared caseload (all care providers know all the patients and work together in the treatment).	Individual care providers responsible for patient assessment and for coordination and treatment.	Individual care providers responsible for patient assessment and for coordination and treatment.
A low caseload (a maximum of ten patients in the team per individual care provider).	A high caseload for the individual practitioner (> 20).	A high caseload for the individual practitioner (> 20).
The care provider takes the initiative on maintaining contacts, and visits patients mainly in their own environment, wherever they are (also when they are hospitalized), the intention being to prevent dropout.	In general, whether contact takes place in the office or at home, involvement ceases (temporarily) after admission has taken place, or if the patient refuses to maintain (long-term) contact. (Normally, there is no contact when the patient is hospitalized.). If patient refuses contact or fails to show up, discharge usually follows.	In general, whether contact takes place in the office or at home, involvement ceases (temporarily) after admission has taken place, or if the patient refuses to maintain (long-term) contact. (Normally, there is no contact when the patient is hospitalized.). If patient refuses contact or fails to show up, discharge usually follows.
Unlimited investment in terms of time (high contact frequency).	Limited contacts, frequency as low as possible.	Limited contacts, frequency as low as possible.
All aid is offered though the ACT team (psychiatric treatment, rehabilitation, assistance with addiction, financial problems, and somatic care).	Only psychiatric care is provided. Addiction, financial problems and other problems are treated by other services.	Only psycho geriatric care is provided. Addiction, financial problems and other problems are treated by other services.
**Disciplines:** Doctor of Medicine or nursing-home doctor (especially for somatic problems) or visiting geriatrist Social worker Psychiatrist Psychologist Community Mental Health Nurse Rehabilitation worker Somatic nurse, Mental Health nurse Homecare worker One of above discipline is specialized in addiction (or double diagnosis)	**Disciplines:** Doctor of Medicine or nursing home doctor Psychiatrist Psychologist, Community Mental Health Nurse	**Disciplines:** Doctor of Medicine or nursing home doctor or visiting geriatrist Psychologist, Community Mental Health Nurse
Each morning there will be a team meeting on all patients in which any necessary appointments are made	Patients are discussed in patient meetings once every six months. Difficult cases are discussed during weekly team meetings.	Patients are discussed in patient meetings once every six months. Difficult cases are discussed during weekly team meetings.
Staff will receive training in ACT methodology	No specific staff training.	No specific staff training.

Table 1 contrasts ACTE working methods with those of CAU [25,26] and [27]), the latter consisting of three teams: a psycho-geriatric team for elderly patients with cognitive problems and two gerontology psychiatric team for elderly patients with psychiatric problems.

Table 1 shows that the main differences between ACTE and CAU will concern team approach including a shared caseload, contact frequency, and the location of care provision. Because care under ACTE will be provided in the patient's living environment, there will often be intensive collaboration with a number of organizations (such as the police in cases of nuisance or homelessness, or with psychiatric hospitals upon admission). Because this group of elderly psychiatric patients is difficult to engage in treatment and may have bad experiences with the care-provision circuit, it is very important than time and energy are devoted to establishing contact with them.

### Outcomes and measurement instruments

Our primary outcome measures will be the number of dropouts (drop-out being defined as no registered contact with the services over a period of 3 months and/or the number of patients discharged out of care). The number of patients who are engaged in care (patients who receive care within 3 months after admission) and patient's psychiatric symptoms, somatic symptoms, and social functioning.

#### Our secondary outcomes measures will be

1. The number of unmet needs,

2. Subjective quality of life,

3. Patients' satisfaction with mental-health care,

4. The number of crisis contacts and (in)voluntary admissions, and the number of admission days. The admission days and crisis contacts were calculated during two periods of observation. The first period was the year before inclusion in the study. The second was the variable observation period ranging till 2 years after inclusion. In both periods, the admission data were standardized as mean number of days per month and crisis contacts as mean number.

#### Assessment instruments

Measurements will be made at three points of the study: at baseline, and after 9 and 18 months. In the meantime, the patients will be treated according to the ACT model or CAU. The same instruments will be used for all three measurements.

The following data will be obtained from the patient's electronic records:

- Demographic and socio-economic characteristics (including gender, age, ethnicity),

- Data concerning living situation, homelessness, employment, and education,

- The psychiatric diagnosis,

- Use of medication,

- Deaths (included suicide) and

- Data on the number of patients who are engaged in care, the number of dropouts and the time between the start of the treatment and dropout. Also the number of crisis contacts and voluntary and involuntary admissions, and the length of stay of admission will be collected.

Using assessment instruments, the following data will be collected:

- Psychosocial functioning (psychiatric, somatic and social symptoms/functioning) by means of the health of nations outcome scale for the elderly (HoNOS 65+, Dutch version) [29,30]

- Care needs by means of the abridged Camberwell assessment of needs elderly (CANE, short version, Dutch version [31,32].

- Quality of life by means of the quality of life (QoL, abridged version of the Manchester short assessment of quality of life; [33,34].

#### HoNOS 65+

The purpose of the HoNOS 65+ [29,30] is to describe the psychological and social functioning of elderly psychiatric patients, and to provide insight into the severity of various psychosocial problems by identifying changes in relevant areas of life.

The HoNOS 65+ can be used to measure the effect of treatment. It consists of 12 items in four sub-scales: behavior disturbance (items 1-3), impairment (items 4-5), symptoms (items 6-8), and social problems (items 9-12). A Dutch addendum to the HoNOS 65+ contains three questions on problems resulting from manic-depressive disorder, motivation for treatment, and medication compliance. These items have not been validated [35]. All items will be completed on a 5-point Likert scale from 0 (no problem), 1 (minor problem), 2 (mild problem), 3 (moderate problem), to 4 (severe problem). The Dutch version of the HoNOS 65+ has been shown to be properly valid [36].

#### Cane

The CANE [31,32] establishes whether an elderly person has problems and/or care needs in various areas of life, and, if necessary, whether adequate effective care is being given. It consists of 26 questions on items such as needing a diet, daily activities, and psychotic symptoms. Care needs are scored in the following manner: 0 (no need), 1 (met need), and 2 (unmet need).

#### QoL

Quality of life will be measured using the QoL scale, which has shown itself to be well suited to measuring satisfaction in the chronic psychiatric target group [33,34]. Dealing with patients' subjective perceptions with respect to quality of life, the scale consists of six elements covering patients' satisfaction with their 1.) life as a whole, 2.) living situation, 3.) personal relationships 4.) physical health, 5.) mental health and 6.) financial situation. Per item, the researcher will ask the patient to award a score on a 7-point Likert scale ranging from 1 (bad or not at all satisfied) to 7 (good and very satisfied).

#### Satisfaction with the care-provider

The QoL scale will be extended with one question on patients' satisfaction with their treatment. This will be taken from the satisfaction questionnaire [34], and, like all answers in the QoL, be rated as a single question on a 7-point Likert scale.

### Other measurement instruments

#### Collaboration between patient and care-provider

The level of collaboration between patient and care provider will be analyzed using the relationship scale from the working relationship questionnaire for case management [37], which consists of seven elements that are filled in by the care provider who is most familiar with the patient. The care provider responsible for the file is the one who is most familiar with the patient. In the CAU this is the patient's clinician. In the ACTE this is the clinician who gets to know the patient best after the intake procedure, and who is also the contact person for family and institutions.

#### Model fidelity of ACT

Research has shown that ACT has the best results when it is implemented in full according to the original ACT model [20,38-44]. As recommended in the literature, model fidelity will be determined on the basis of both conditions [20].

The fidelity of ACT and CAU will be measured twice (in 2010 and in 2011) by two independent researchers using the Dartmouth assertive community treatment scale (DACTS). The DACTS consists of 28 items covering three dimensions (team structure, organizational structure, and features of service delivery). All twenty-eight items are rated on a 5-point scale range from 1 (no implementation or only a low degree of implementation) to 5 (complete implementation) [45-47].

Table 2 shows an overview of all outcome measures and instruments.

**Table 2:** Overview of outcome measurements

Variable	Instrument	Assessed by	Baseline	9 month	18 month
Socio-demographics	Patient records	Interviewer	X	X	X
Psychiatric history	Patient records	Psychiatrist or doctor	X		
Psychiatric	DSM IV Patient	Psychiatrist or	X	X	X
diagnosis	records	doctor			
Drop-out	Registration system	Researcher	X	X	X
Crisis contacts	Registration system	Researcher	X	X	X
Admission days	Registration system	Researcher	X	X	X
Psychosocial functioning	HoNOS 65+	Interviewer	X	X	X
Care need	CANE short version	Interviewer	X	X	X
Quality of life	QoL	Interviewer	X	X	X
Satisfaction with care	One question on 7- point Likert scale	Interviewer	X	X	X
Collaboration between patient and care-provider	Working Relationship Questionnaire for Case Management	Care provider	X	X	X
ACT model fidelity	DACTS	Trained ACT evaluators	X		X

### Power analysis

The sample size was determined by means of a power analysis. To detect a clinically significant impact (average effect size (Cohen's d) ≥ 0. 6) on the primary outcome variable (number of patients remain in care with an alpha of 0. 05 and a power of 0.85), we needed at least 50 patients per condition. In another ACT trial, Sytema et al [48] found 20% lose for randomization due to patients' failure to meet the inclusion criteria, and patients who refused to give informed consent for the interview [48]. We therefore wanted to include 80 patients in the ACTE team and 80 patients in the CAU condition. This will be enough to prove a clinically significant effect.

### Randomization

The patients were referred to BavoEuropoort by general practitioners or other institutions (such as the police or municipal health service), who filled out a form describing the patient's characteristics and current problems. To determine whether the patients fulfilled the inclusion criteria, this form was reviewed using a checklist by the researcher (JS) and clinicians. If the patient did meet the criteria, he or she was allocated a number chronologically by the service administrator. Each number was given to the researcher, who then decided on the condition (intervention or care as usual). This was done on the basis of a previously arranged list of numbers randomized to each of the two conditions (with help from http://www.randomizer.org). To ensure similar numbers in both conditions the pre-arranged list of numbers are based on a block design (block size of four). Inclusion and randomization of patients was done before informed consent was obtained for participation in an interview with the interviewer.

### Statistical analysis

Analyses will be performed according to the *intention to treat *principle, which means that everyone who is randomized stays in the group in which they were randomized. Statistical package for the social sciences, version 15.0 will be used to analyze the data. The primary outcome measure is the differences between ACTE and CAU regarding the number of patients who were out-of-contact with mental health services. Secondary outcome measures are improvement of psychosocial functioning (total HoNOS 65+ score, total score from baseline to the final interview after 18 months), need-for-care over time (primary outcome measures), the number of crisis contacts and (in)voluntary admissions, and the number of admission days (these last 3 measures will be retrieved from administration files) The interviewer collects the other secondary outcome measures (subjective quality of life and client satisfaction.) Categorical variables shall be analyzed using chi-square tests. Mixed models with repeated measurement were applied for the continuous outcomes. This model will be used because of the ability to include participants with missing data. Missing data at the second and third interview will be imputed with the last carried out interview.

### Ethical considerations

Because the patients included in the study are difficult to engage, they will be allocated randomly over the two conditions without asking informed consent. This Zelen design [24] and the execution of this study were approved by the Dutch union of medical-ethic trial committees for mental health organizations. Data will be processed anonymously. Confidential information and patient names will be treated according to the medical confidentiality rules. All personal details will be awarded a code to which only the research team has the key.

### Research procedures & timelines

This study will proceed in four stages:

a. Preparation for data collection (1 March 2008 to 1 July 2008)

b. Inclusion of patients (1 July 2008 to 1 August 2010)

c. Follow-up (1 April 2009 to 1 March 2012)

d. Data-analysis, publication of articles, knowledge transfer, and writing of a thesis (1 March 2012 to 1 March 2013).

## Discussion

The central research question in this study is whether ACTE can ensure that elderly people with SMI remain in care and whether ACTE can reduce psychosocial symptoms and unmet needs more than CAU. Other research questions include whether the intervention can reduce the number of crisis contacts, reduce the number and duration of voluntary and involuntary admissions, and improve quality of life and client satisfaction.

This study will thus provide opportunities to examine how well ACTE works in practice with this group of elderly people, and whether it has any extra value over CAU. By helping to increase overall understanding of various forms of mental healthcare, its implementation may lead to an evidence-based intervention for this special group. If ACTE is shown to have added value, it can be implemented, and further research can follow.

The study results will be prepared for national and international publication and for presentation at national and international conferences. They may also lead to the publication of a handbook on ACT for the elderly.

This study has two specific strengths and two limitations. The first strength is that we will use and RCT design to test the effectiveness of ACT for the elderly. The second is its internal validity, which is protected by the structured protocol monitoring the ACT model, as well assessing the contents of the CAU by also applying the DACTS scale.

The first limitation is the fact that community mental healthcare professionals and the independent research assessor will not be blinded for which patients are included in this study. Because the only way in which independent research assessor can reach elderly people with SMI is through collaboration with the community mental healthcare professionals, it will not be possible to blind them both. The second limitation is that CAU exists of different kind of teams in different area, with different working methods. These working methods will be describe in the outcome of the DACTS but can negatively or positively affect the outcome results. In addition, the effects of ACTE will also depend on the CAU. When the quality of care of the CAU is high, there is less chance of demonstrating an effect of ACTE. This limitation is a problem in all studies comparing a new intervention with CAU [49]. Therefore, we will describe CAU thoroughly also using the DACTS, to demonstrate in what aspects ACTE and CAU differ.

The last limitation is the inclusion format. We started with the following inclusion criteria: (1) age 65 year or older; (2) having a severe mental illness, (3) no motivation for treatment, and (4) having four or more additional problems within the following domains: addiction, somatic problems, activities of daily living, housing, daytime activities, social relationships, finances, or police contacts. Untill June 2009 only 35 patients were included. Therefore we broadened our inclusion criteria by lowering the minimum age to 60 years. January 2010 only 50 patients were enrolled and we decided to limit the number of problems in several domains to 2 (was 4). Finally, in March 2010 we included only 56 patients, and then we let loose of criterion no 4. At the end of the trial by July 31 2010 we recruited 64 participants. Till August 1^st ^2010, fife patients died, 3 in the intervention group and 2 in the control group (no suicides). Because of the number of patients enrolled in this study (64 patients) the power of the study is a problem.

## Competing interests

The authors declare that they have no competing interests.

## Authors' contributions

JS constructed the design of the study and drafted this paper. All the authors revised the study protocol. CLM revised this manuscript. All authors read and approved the final manuscript.

## List of abbreviation used

ACT: Assertive Community Treatment; ACTE: Assertive Community Treatment for the elderly; CANE: Camberwell Assessment of Needs Elderly; CAU: Care as Usual; DACTS: Dartmouth Assertive Community Treatment Scale; HoNOS: Health Of the Nation Outcome Scale; QoL: Quality of Life; RCT: Randomized Controlled Trial; SMI: Severe Mental illnesses.

## Pre-publication history

The pre-publication history for this paper can be accessed here:

http://www.biomedcentral.com/1471-244X/10/84/prepub
